# Research on Classification Algorithm of Silicon Single-Crystal Growth Temperature Gradient Trend Based on Multi-Level Feature Fusion

**DOI:** 10.3390/s24041254

**Published:** 2024-02-15

**Authors:** Yu-Yu Liu, Ling-Xia Mu, Peng-Ju Zhang, Ding Liu

**Affiliations:** 1 School of Automation and Information Engineering, Xi’an University of Technology, Xi’an 710048, China; liuyy@stu.xaut.edu.cn (Y.-Y.L.);; 2 Shaanxi Key Laboratory of Complex System Control and Intelligent Information Processing, Xi’an University of Technology, Xi’an 710048, China

**Keywords:** entropy, multi-level feature fusion, crystal growth, DBN

## Abstract

In the process of silicon single-crystal preparation, the timely identification and adjustment of abnormal conditions are crucial. Failure to promptly detect and resolve issues may result in a substandard silicon crystal product quality or even crystal pulling failure. Therefore, the early identification of abnormal furnace conditions is essential for ensuring the preparation of perfect silicon single crystals. Additionally, since the thermal field is the fundamental driving force for stable crystal growth and the primary assurance of crystal quality, this paper proposes a silicon single-crystal growth temperature gradient trend classification algorithm based on multi-level feature fusion. The aim is to accurately identify temperature gradient changes during silicon crystal growth, in order to promptly react to early growth failures and ensure the stable growth of high-quality silicon single crystals to meet industrial production requirements. The algorithm first divides the temperature gradient trend into reasonable categories based on expert knowledge and qualitative analysis methods. Then, it fuses the original features of actual production data, shallow features extracted based on statistical information, and deep features extracted through deep learning. During the fusion process, the algorithm considers the impact of different features on the target variable and calculates mutual information based on the difference between information entropy and conditional entropy, ultimately using mutual information for feature weighting. Subsequently, the fused multi-level feature vectors and their corresponding trend labels are input into a Deep Belief Network (DBN) model to capture process dynamics and classify trend changes. Finally, the experimental results demonstrate that the proposed algorithm can effectively predict the changing trend of thermal field temperature gradients. The introduction of this algorithm will help improve the accuracy of fault trend prediction in silicon single-crystal preparation, thereby minimizing product quality issues and production interruptions caused by abnormal conditions.

## 1. Introduction

Integrated circuits are the cornerstone of modern technology, the driving force behind the booming information industry, and an important reflection of national strength [1]. Nowadays, semiconductor silicon single crystals are widely used in many fields of the national economy and defense technology. The Czochralski method (CZ) is the mainstream method for growing silicon single crystals [2]. With the rapid development of the information technology industry and the continuous improvement in the linewidth requirements of large-scale integrated circuits, silicon single-crystal growth is moving towards large size and electronic grade. However, with the continuous improvement in quality requirements for crystal growth, crystal growth equipment is becoming increasingly complex. The design, manufacturing, testing, operation, and maintenance costs throughout the lifecycle of the equipment have increased significantly. At the same time, the probability of equipment failure, performance degradation, and functional failure also increases, thereby affecting the safety and crystal quality of the silicon single-crystal growth process. A stable thermal environment is the primary guarantee for producing high-quality silicon single crystals, and the temperature gradient near the solid–liquid interface is generally related to the crystal diameter [3,4]. However, due to limitations in production equipment and detection technology, it is difficult to directly obtain temperature gradients, and the number of temperature measurement points in the thermal field is limited, with significant process lag. Therefore, it is usually necessary to carefully design experiments and conduct multiple repetitions to explore the process curve, which is time-consuming and costly. Therefore, research on the real-time monitoring of temperature gradient trends in the thermal field is necessary. There are two reasons for this:

1. The change in temperature gradient indeed provides information about the local and instantaneous temperature distribution. The temperature gradient, which represents the rate of temperature change between a point in the thermal field and its neighboring points, is a physical quantity that measures the temperature variation within a unit distance. This concept reveals the direction and rate of the most rapid temperature change in a specific area. However, relying solely on the information of instantaneous temperature gradient changes to take control measures may lead to inappropriate adjustments and increased furnace condition fluctuations. This is because the temperature gradient at a given moment may change suddenly but can be corrected in the next moment.
2. The change trend of the thermal field temperature gradient serves as the basis for identifying early abnormal states. The operation and control of the silicon single-crystal growth process primarily rely on accurately classifying the change trend of the temperature gradient.

In actual production, on-site operators primarily rely on manual experience to judge the change trend of the temperature gradient, which makes it difficult to ensure the quality of the crystals. However, this method has certain uncertainties regarding the reliability of production. In response to the aforementioned issues, there is an increasing demand in the industry for the real-time monitoring of the crystal growth process. This study aims to establish a classification model for the trend of temperature gradient changes in the CZ silicon single-crystal growth process and apply it to practical work. Currently, the monitoring of production status in industrial production processes is commonly achieved through mechanism-based methods, data-based methods, and expert experience [5,6]. In fact, the silicon single-crystal growth process is characterized by severe parameter coupling, high nonlinearity, strict requirements for parameter fluctuations, high safety and reliability requirements, unclear mechanisms, and difficulties in establishing mathematical models. However, the silicon single-crystal growth process is severely coupled, highly nonlinear, requires strict parameter stability, and has high requirements for safety and reliability. Additionally, the mechanism is not clear, and it is difficult to establish mathematical models. Therefore, implementing mechanism-based methods is challenging [3]. At the same time, with the development and application of distributed control systems and the rapid advancement of storage technology, a large amount of data can be collected during the silicon single-crystal growth process. Therefore, it is more reasonable to apply data-driven techniques to classify the trend of temperature gradient changes in the thermal field.

Data-driven modeling techniques have been widely used in the industry after decades of development, mainly to solve modeling problems related to key indicators in industrial processes [7,8,9]. These techniques include Support Vector Machines, partial least squares, neural networks, and deep learning networks. Wan et al. [10] studied soft measurement modeling methods for the V/G ratio in the CZ silicon single-crystal growth process using a stacked autoencoder network. Zhang et al. [3] combined the finite element method (FEM) with control strategies to study the regulation of crystal growth processes. This control strategy solves the problem of controlling crystal temperature in traditional crystal growth systems and enables the growth of high-quality single-crystal silicon. Ren et al. [11] proposed a modeling method for soft sensors called VMD-SEAE-TL. This method combines variational mode decomposition (VMD), stacked enhanced autoencoder (SEAE), and transfer learning (TL) algorithms, aiming to detect key variables in the industrial production process of silicon single crystals. Due to the complexity of silicon single-crystal production, predicting key indicators is challenging.

Most of the existing prediction models for key indicators are built on supervised learning algorithms using labeled data. However, in practical applications, the inconsistent sampling frequency of process variables leads to a waste of a large amount of unlabeled data, resulting in a “data-rich, information-poor” situation. Secondly, these models usually only use single-layer features to describe the silicon single-crystal growth process. Therefore, when operating conditions fluctuate, especially after long-term operation, the accuracy of the model may deviate [12]. In response to this issue, researchers have proposed a new adaptive nonlinear predictive control method for crystal diameter based on hybrid integration modeling [13,14]. This method combines engineering practice and decomposes the original data into several sub-sequences using the wavelet packet decomposition method. It removes high-frequency signals from the sub-sequences to reduce non-stationarity and the impact of random noise. Next, each sub-sequence is modeled separately, and the prediction results of each model are fused. Compared to a single model, this integrated modeling approach significantly improves the prediction accuracy. Furthermore, the prediction results of this method mainly rely on current information, but the models constructed usually cannot accurately describe the entire process of silicon single-crystal growth. Inspired by the above content, this study uses multi-level features to describe the complex silicon single-crystal growth process. Among them, the classification period of the temperature gradient trend model should not be too short, because if only the time interval between two offline analysis values is considered, then this short-term local information provides limited decision support for onsite operators. Therefore, this paper determines the classification time interval of the trend change based on expert experience and data analysis. In addition, due to the advantages of DBN in effectively describing object characteristics and their achievements in multiple fields, we choose to use DBN networks to establish the model.

This study establishes a data-driven model based on multi-level feature fusion to classify the change trends of variables. The main contributions of this paper are as follows:

1. For the CZ silicon single-crystal growth process, this paper proposes a pioneering data-driven method for monitoring the change trend of the thermal field temperature gradient, which is used to monitor the real-time changes in temperature gradient. This method lays the foundation for the safety and quality control of the silicon single-crystal growth process. This marks the first application of process monitoring in this field.
2. To obtain appropriate temperature change trend labels, we designed a method based on expert knowledge and qualitative analysis to determine the classification time interval so that the model can provide results with more decision support. Next, we qualitatively defined trend labels through different basic elements, which can comprehensively describe the growth status of silicon single crystals.
3. To ensure the accuracy of the model under fluctuating operating conditions, this paper employs multiple methods to extract raw features, shallow features, and abstract features to describe the process of silicon single-crystal growth. Specifically, we use variable analysis, statistical information, and SAE to extract these three types of features. Each type of feature corresponds to a different description of the same stage.

The remaining content is arranged as follows. First, Section 2 briefly introduces the basic theory of SAE and DBN models. Then, in Section 3, we propose a basic research framework, which includes feature extraction, obtaining silicon content trend labels, and multi-level feature fusion. Next, in Section 4, we conduct simulation experiments and analyze the effectiveness of the multi-level fusion feature algorithm. Finally, in Section 5, we summarize the work performed in this article and provide prospects for future research directions.

## 2. Preliminaries of SAE and DBN

### 2.1. SAE

**Figure 1:**
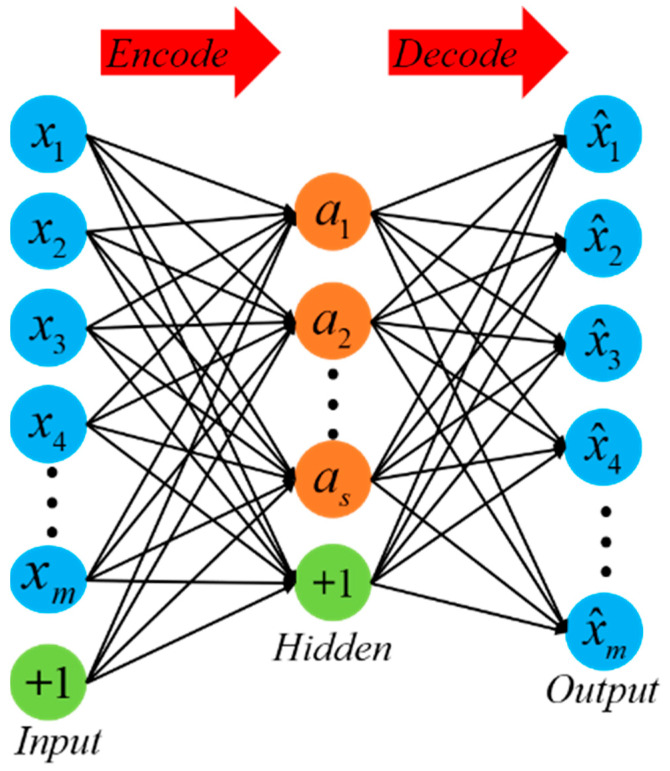
Autoencoder structure.

An autoencoder (AE) is an unsupervised learning model that is trained using the backpropagation algorithm and optimization methods such as gradient descent [11,12]. Its working principle is to utilize the input data for supervision and attempt to learn a mapping relationship in order to obtain a reconstructed output [15]. A typical autoencoder consists of a simple three-layer network, including an input layer, a hidden layer, and an output layer. The mapping process from the input layer to the hidden layer is considered the encoding process, while the mapping process from the hidden layer to the output layer is equivalent to the decoding process, aiming to reconstruct the original input as closely as possible, as shown in Figure 1.

**Figure 2:**
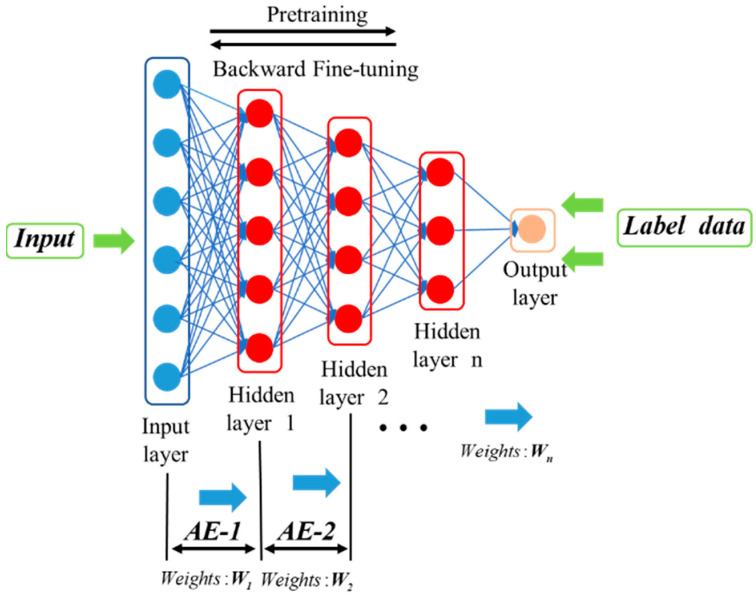
Stacked autoencoder network structure.

The stacked autoencoder (SAE) is a network structure constructed by stacking multiple such autoencoders, as shown in Figure 2. This network structure is mainly used to extract deep data features from massive industrial data.

The encoding and decoding processes of an AE network are shown as (1) and (2), respectively:

(1) $$a=s_{f}(W_{1}x+b_{1})$$

(2) $$\hat{x}=s_{d}(W_{2}a+b_{2})$$

where the network input is denoted as x and the reconstructed output is denoted as $\hat{x}$. In both processes, we use the sigmoid function, represented as $s_{f}$ and $s_{d}$, as the activation function for the neurons. The features of the hidden layer are represented as a, and the weights for the encoding and decoding processes are denoted as $W_{1}$ and $W_{2}$, respectively. The biases for the encoding and decoding processes are denoted as $b_{1}$ and $b_{2}$, respectively.

The main objective of an AE network is to achieve input reconstruction by minimizing the reconstruction error. The loss function for this purpose is shown as (3):

(3) $$J(W_{1},W_{2},b_{1},b_{2})=\frac{1}{m}\sum_{i=1}^{m}{\left(x_{i}-{\hat{x}}_{i}\right)}^{2}$$

When multiple autoencoders are stacked layer by layer, it forms an SAE network. The SAE is an unsupervised learning network. In order to achieve supervised learning for soft sensor modeling, the decoding process of the stacked autoencoders is removed, and an output layer is added at the end to predict the target variable.

### 2.2. DBN

**Figure 3:**
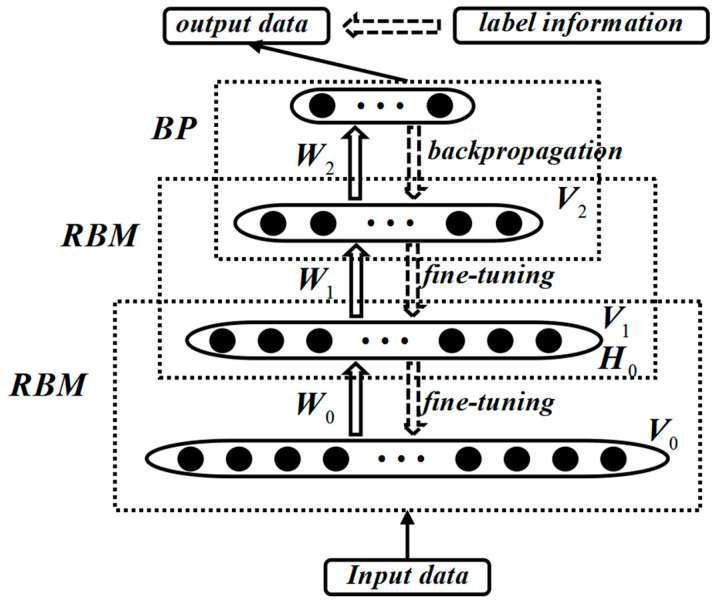
Typical DBN structure.

The DBN is a probabilistic generative model proposed by Geoffrey Hinton in 2006 [16]. It is primarily composed of several Restricted Boltzmann Machine (RBM) units and a stacked error backpropagation network (BP), forming a deep neural network structure. The connections between neurons in the DBN form a graphical structure, which can generate training data based on the maximum likelihood [17]. From the perspective of structural composition, the DBN structure is shown in Figure 3. In the training process of the DBN, a layer-wise training strategy is adopted, which effectively addresses the training challenges of deep neural networks. DBNs can be used not only for unsupervised learning but also for supervised learning, making it widely applicable in practical applications.

The RBM is an efficient unsupervised learning model that is primarily used for effectively extracting data features and constructing new data structures for predictive analysis [18]. In neural networks, the RBM is often used as a part of initializing a feedforward neural network to improve the network’s generalization ability.

**Figure 4:**
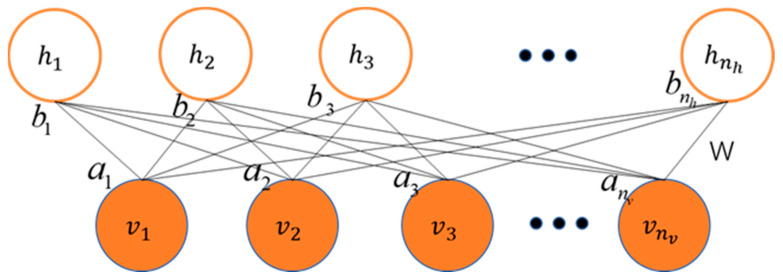
RBM network structure diagram.

From the perspective of network structure, as shown in Figure 4, the RBM is a shallow neural network with two layers, including a visible layer and a hidden layer. The visible layer, also known as the input layer, consists of visible units and is used to input training data. The hidden layer consists of hidden units and can be seen as feature detectors. Unlike Boltzmann Machines (BMs), the RBM requires no connections between neurons within the same layer. In other words, when the state of visible layer neurons is given, the activation of hidden layer neurons is independent, and vice versa. Therefore, RBMs can learn different features at different levels. It is worth mentioning that the RBM, due to its excellent feature extraction capability, is often used as a learning module in DBNs. The DBN is a deep neural network composed of multiple stacked RBMs. In this way, DBNs can learn increasingly abstract and advanced features at different levels.

As shown in the Figure 4, $v={\left(v_{1},v_{2},\cdots ,v_{i},\cdots v_{m}\right)}^{T}\;\;\;i,m\in Z^{*}$ represents the state vector of the visible layer, $v_{i}$ represents the value of the *i*-th node in the visible layer, and m is the number of nodes in the visible layer. $h={\left(h_{1},h_{2},\cdots ,h_{j},\cdots h_{n}\right)}^{T},\;j,n\in Z^{*}$ represents the state vector of the hidden layer, $h_{j}$ represents the value of the j-th node in the hidden layer, and n is the number of nodes in the hidden layer. $W=\left(w_{ij}\right),\;i\in \left[1,m\right],\;j\in \left[1,n\right]$ represents the weight matrix, $a={\left(a_{1},a_{2},\cdots ,a_{i},\cdots a_{m}\right)}^{T}$ represents the bias vector of the visible layer, and $b={\left(b_{1},b_{2},\cdots ,b_{j},\cdots b_{n}\right)}^{T}$ represents the bias vector of the hidden layer. $\theta =\left(W,a,b\right)$ represents the parameters in the RBM, for any *i* and *j*, $v_{i},h_{j}\in \left\{0,1\right\}$.

RBM can also be viewed as an energy-based model, where the network’s equilibrium corresponds to the lowest-energy state. For a given set of $\left(v,h\right)$, the specific energy function can be represented as follows:

(4) $$E_{\theta }\left(v,h\right)=-\sum_{i=1}^{m}a_{i}v_{i}-\sum_{j=1}^{n}b_{j}h_{j}-\sum_{i=1}^{m}\sum_{j=1}^{n}h_{j}w_{ij}v_{i}$$

The joint distribution of the RBM defined by the energy function is

(5) $$P\left(v,h\right)=\frac{1}{Z}e^{-E\left(v,h\right)}$$

where $Z=\sum_{v,h}e^{-E\left(v,h\right)}$ is the normalization factor to ensure that the sum of the probability distribution is 1. By taking the marginal distribution of the joint probability distribution, we can obtain:

(6) $$P\left(v\right)=\sum_{h}\frac{1}{Z}e^{-E\left(v,h\right)}$$

(7) $$P\left(h\right)=\sum_{v}\frac{1}{Z}e^{-E\left(v,h\right)}$$

The training process of DBNs mainly consists of two stages: pre-training and fine-tuning. In the pre-training stage, each layer of the RBM network undergoes individual unsupervised training to initialize weights and extract features. The goal of this stage is to ensure that the feature vectors can map to different feature spaces and preserve the feature information of the original data as much as possible. Specifically, the pre-training process is conducted through unsupervised greedy layer-wise training. It starts from the bottom by training the bottommost RBM with the original input data and then uses the features extracted by the bottom RBM as input to train the top RBM. This process is repeated to train as many RBM layers as possible.

After completing pre-training, the DBN enters the fine-tuning stage. In this stage, a backpropagation neural network (BPNN) is set up at the last layer of the DBN. The output of the last RBM is used as input to the BPNN, and the entire network is optimized through supervised learning to further adjust the parameters and improve the model’s performance. The purpose of fine-tuning is to optimize the data feature extraction capability of the DBN, making it better suited for various tasks such as classification, regression, feature learning, and transfer learning [19,20].

## 3. Classification Model Based on Multi-Level Feature Fusion

The data-driven classification method introduced in this section is based on multi-level feature fusion and the Deep Belief Network (DBN). The method is mainly divided into four parts: acquiring temperature gradient trend labels, deep feature extraction, multi-level feature fusion, and overall classification framework.

In the temperature gradient trend label acquisition stage, temperature gradient trend labels for different stages of silicon single-crystal growth are obtained through expert qualitative analysis. These labels effectively describe the different stages of the growth process and provide important auxiliary information for subsequent classification tasks. Then, the deep feature extraction stage is where we use unsupervised learning methods to extract temperature gradient features from the silicon single-crystal growth process. These features fully reflect various information about the growth process and provide a foundation for subsequent feature fusion and classification. Then, the multi-level feature fusion stage is where we fuse features from different levels to fully utilize various feature information. Lastly, the overall classification framework involves inputting the extracted features from different levels into the DBN for training and testing, thus achieving automatic classification of the silicon single-crystal growth process.

### 3.1. Labeling Temperature Gradient Trend Changes

**Figure 5:**
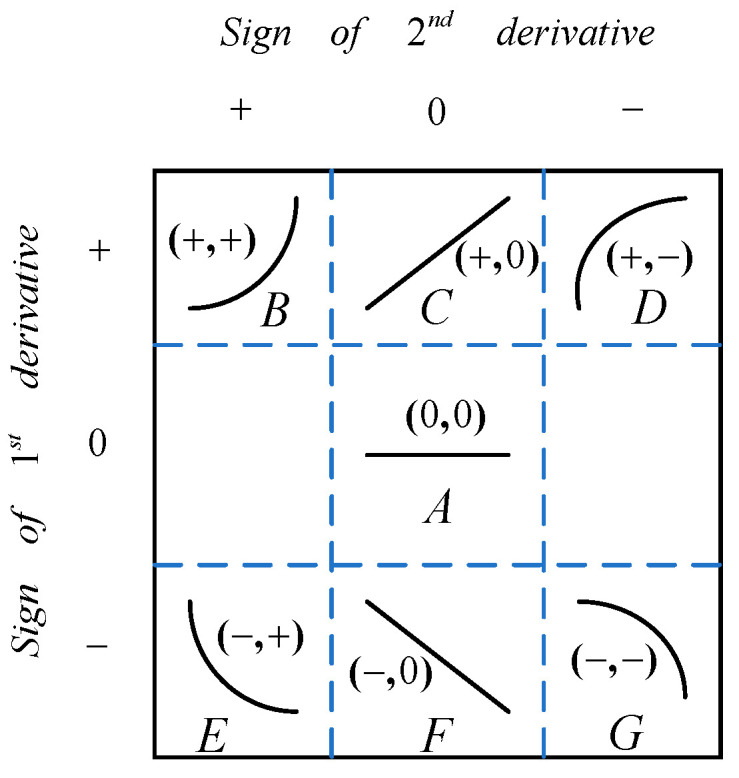
Shapes of seven primitives.

In the process of preparing silicon single crystals, failure to promptly detect and adjust abnormal conditions can lead to unexpected product outcomes or even crystal pulling failure. Therefore, accurately identifying abnormal furnace conditions is crucial for producing perfect silicon single crystals. The most direct manifestation of changes in the growth conditions of silicon single crystals is the temporal variation in the thermal field temperature gradient, which exhibits certain trends on the time axis. Recognizing these trend patterns helps identify early abnormal situations and provides guidance for on-site operations [18]. The entire process of silicon single-crystal growth typically lasts for over 100 h, and its fundamental requirement is stable operation. If control is based on transient changes in the thermal field temperature gradient, it can result in improper adjustments and frequent fluctuations in furnace conditions. On the other hand, if control is based on long-term changes in the thermal field temperature gradient, it may lead to a decline in crystal quality or crystal variation. Additionally, there is a time delay of approximately 11–30 min in adjusting the power of the heater. Taking all these factors into consideration, expert experience suggests choosing a time interval of 20 min to classify the change trends of the thermal field temperature gradient. The classification results are shown in Figure 5.

The seven trend primitives are represented by first-order and second-order derivatives. They are denoted as A(0,0), B(+,+), C(+,0), D(+,−), E(−,+), F(−,0), and G(−,−). A sliding window is used to group the thermal field temperature gradient on the time axis. The data within the sliding window are fitted using polynomial regression, and the signs of the first-order and second-order derivatives of the fitting equation describe the trend of the thermal field temperature gradient changes. This method qualitatively describes the temperature trend information using the seven primitives (Algorithm 1).
**Algorithm 1:** Labeling the Trend of Key Process Indicators    **Input:** Values of thermal field temperature gradient, size of sliding window.    **Output:** Trend labels.    **Begin:**    Step 1: Determine the total window width M, where $\left(\left(x_{1},y_{1}\right),\left(x_{2},y_{2}\right),...,\left(x_{M},y_{M}\right)\right)$ is predetermined in the study. Based on the classification time interval, determine the width of the sliding window as 20 min.    Step 2: Fit the data in the sliding window using a zero-order polynomial. Determine the goodness of fit using an F-test. If the equation is not significant, record the trend of thermal field temperature gradient changes as stable. Otherwise, proceed to Step 3.    Step 3: Fit the data using a first-order polynomial and determine the goodness of fit using an F-test. If the equation is not significant, determine the trend as linear increasing or linear decreasing based on the sign of the slope. Otherwise, proceed to Step 4.     Step 4: Fit the data using a second-order polynomial. If the first-order and second-order derivatives are positive, it is considered a convex upward trend. If the first-order derivative is positive and the second-order derivative is negative, it is considered a concave upward trend. If the first-order derivative is negative and the second-order derivative is positive, it is considered a convex downward trend. If both the first-order and second-order derivatives are negative, it is considered a concave downward trend. If the first-order and second-order derivatives do not fall into the above cases, proceed to step 5.    Step 5: Select the equation with the best fit and record the trend information.    Step 6: Perform Steps 2 to 5 for each sliding window to obtain the trends. The termination condition is when all sliding windows have been processed appropriately.    **End**

### 3.2. Deep Feature Extraction

**Figure 6:**
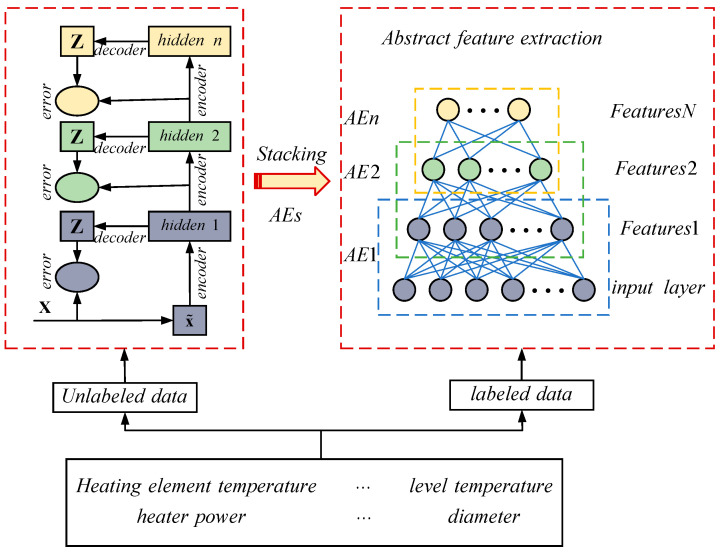
Block diagram of deep abstract feature extraction.

The SAE is a method that learns abstract features layer by layer, allowing for the better representation of complex systems [21,22]. This concept has gained widespread attention and has achieved state-of-the-art results in many fields. Furthermore, the SAE is constructed based on greedy layer-wise unsupervised pre-training, making it particularly suitable for situations where there is a lack of labeled samples due to inconsistent sampling frequencies of industrial sensors. In this study, the SAE is used to extract abstract features, and the implementation details are shown in Figure 6.

A deep network is composed of numerous independently functioning autoencoders within a deep architecture. The training process of the autoencoders is similar to that of traditional neural networks. The model parameters are updated by randomly setting weights and biases and utilizing error backpropagation. The unique aspect is that the input and output of the model are the same. After training the (n − 1)th autoencoder with the original data, the output of its hidden layer serves as input for training the nth autoencoder, and this process continues in a cycle.

During the pre-training phase, there is no specific task, and the focus is on unsupervised feature extraction from unlabeled data. After layer-wise pre-training is completed, all the hidden layers are combined to construct the deep network. The network parameters are based on the learned parameters during the pre-training process [23].

### 3.3. Multi-Level Feature Fusion Based on Mutual Information

The growth of silicon single crystals by the CZ method is a highly complex process that cannot be accurately described by abstract features. Based on the expertise and process knowledge of silicon single-crystal growth, three shallow features are constructed to reflect the trend of changes within a certain period of time (20 min). In the growth process of silicon single crystals, the range of variation (R), the rate of variation (∆x/∆t), and the standard deviation (S) are key features that reflect their developmental trends. The following are the formulas for calculating these features:

(8) $$R=x_{\max}-x_{\min}$$

(9) $$\Delta x/\Delta t=\frac{1}{n}\left[\sum_{i=n+1}^{2n}x_{i}-\sum_{i=1}^{n}x_{i}\right]$$

(10) $$s=\sqrt{\frac{1}{n-1}\sum_{i=1}^{n}{\left(x_{i}-\bar{x}\right)}^{2}}$$

where $x_{\max}$ and $x_{\min}$ represent the maximum and minimum values of the variable, $x_{i}$ represents the *i*-th value of the variable, n represents the number of values within the 20 min period, and $\bar{x}$ represents the mean value of the variable. These shallow features describe the range of fluctuations and the degree of dispersion of the process variables, which can reflect the state of the thermal field in the silicon single-crystal furnace within the 20 min period. By analyzing these features, we can understand the changes in the thermal field during the silicon single-crystal growth process, providing insights for optimizing thermal field design and improving crystal quality.

In the classification model, the input is divided into three main parts: raw features collected by sensors, shallow features extracted based on statistical information, and abstract features learned through deep networks. After considering the impact of different features on the target variable, a feature weighting method based on mutual information is proposed [22]. Let us assume that the dataset X has L different classes, where L represents seven different sets of trend information. The dataset corresponding to the *i*-th class is denoted as $x^{i}$, i=1,2,⋯,L, and $N_{X}$ and $N_{x^{i}}$ represent the number of features X and $x^{i}$, respectively. The information entropy of the entire dataset is calculated as follows (for the *i*-th class in the entire dataset):

(11) $$Info(X)=-\sum_{i=1}^{L}\frac{N_{x^{i}}}{N_{X}}\log_{2}\left(\frac{N_{x^{i}}}{N_{X}}\right)$$

Let us assume the dataset has n features as $\left\{x_{1},\cdots ,x_{k},\cdots ,x_{n}\right\}$. Each feature is a t-dimensional vector $x_{k}=(x_{k}^{1},x_{k}^{2},\cdots ,x_{k}^{t})$. The dataset is divided into L categories (categories representing the trend of changes), denoted as $\left(X^{1},X^{2},\cdots ,X^{t}\right)$. The formula for calculating the conditional entropy of a feature $x_{k}$ is as follows (for a specific feature belonging to a certain class in the entire dataset):

(12) $$Info_{x_{k}}(X)=-\sum_{j=1}^{t}\frac{N_{X^{j}}}{N_{X}}\times Info(X^{j})$$

The mutual information of feature $x_{k}$ with respect to category $G_{x_{k}}$ can be described as follows:

(13) $$G_{x_{k}}=Info(X)-Info_{x_{k}}(X)$$

If we obtain the mutual information for n different features, then the weight $\left\{w_{1},\cdots ,w_{k},\cdots ,w_{n}\right\}$ of the feature vector $\left\{x_{1},\cdots ,x_{k},\cdots ,x_{n}\right\}$ can be calculated as follows:

(14) $$w_{i}=G_{x_{i}}/\sum_{i=1}^{n}G_{x_{i}}$$

**Figure 7:**
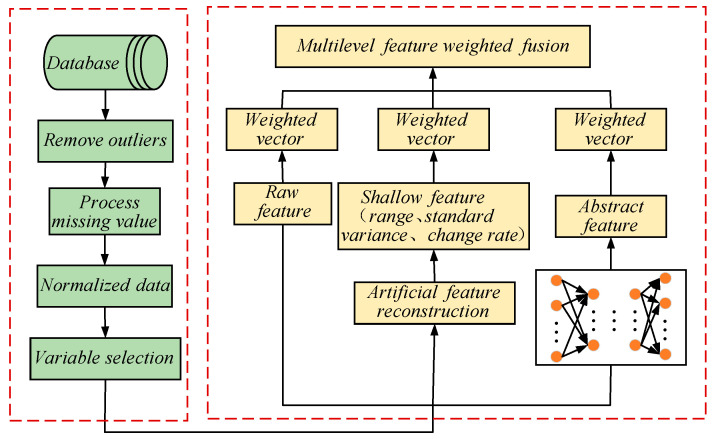
Diagram of the multi-level feature fusion process based on mutual information.

Based on mutual information, we aim to fuse multi-level features to compensate for the shortcomings of single features. Through this method, features closely related to the target variable are strengthened, while those unrelated to it are weakened [24]. Figure 7 shows the ingenious process of multi-level feature fusion.

### 3.4. Classification of Silicon Content Trend Based on Multi-Level Feature Fusion with DBN

This study focuses on classifying trend changes in a multi-class problem. The method is used to directly input the output of the hidden layers of a DBN into a softmax layer. For the data collected from multiple sensors, mutual information is used for multi-level feature fusion. The corresponding labels of thermal field temperature gradient trends and the fused features are input into the DBN model for training, resulting in the final classification results (Algorithm 2).
**Algorithm 2:** Multi-level Feature Fusion Model for Classification    **Input:** X, Y, dataset, SAE parameters, DBN parameters, maximum number of iterations, maximum number of hidden layers.    **Output:** Classification results for the test samples.    **Begin:**Based on expert experience and theoretical analysis, select data from the historical database for modeling.Preprocess the data, obtain the raw features through correlation analysis, and divide the dataset into training and testing sets.Using human expertise to extract shallow features while using SAE networks to learn deep features of the data.After considering the impact of different features on the target variable, perform feature weighting.Divide the temperature gradient time series into segments and extract trend information through polynomial regression fitting.Train the DBN model using the fused features and the corresponding temperature gradient trend labels, and obtain the optimal parameters through the sparrow optimization algorithm.Based on the multi-level fusion model, the classification results are obtained, and the model accuracy is evaluated using the test set.    **End**

## 4. Experimental Verification

### 4.1. Experimental Data Description and Preprocessing

The historical database of the silicon single-crystal growth process is an important data source that reflects the process state information. In this study, actual production data from a specific model of a single-crystal furnace were selected to train the model, and Table 1 provides a detailed description of the variables. However, due to equipment failures and human errors, there may be outliers in the data. Preprocessing the data is necessary to improve the quality and accuracy of the classification model’s data processing. The box plot method is used to remove outliers, as it accurately describes the data distribution and quickly identifies outliers. Since the dataset is large, any missing data in the database are directly deleted [25]. After the aforementioned preprocessing, we scale the values to the range of [0, 1]:

(15) $$x_{norm}=\left(x_{i}-x_{\min}\right)/\left(x_{\max}-x_{\min}\right)$$

where $x_{norm}$ represents the result of variable normalization. $x_{\max}$ and $x_{\min}$, respectively, represent the maximum and minimum values of the *i*th variable.

**Figure 8:**
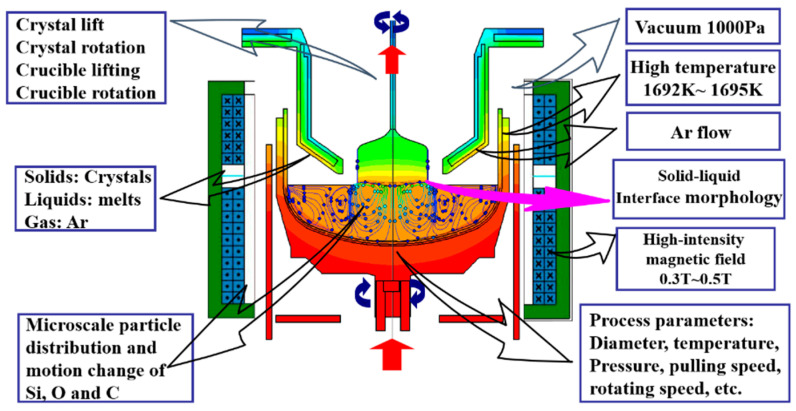
A schematic diagram of the CZ silicon single-crystal growth environment.

**Figure 9:**
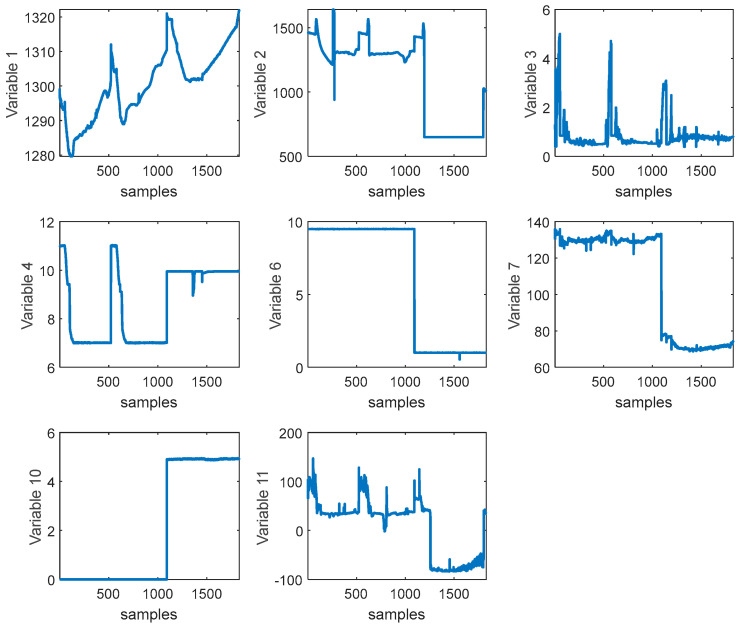
Raw data for variable screening.

Considering the complexity of the crystal growth environment, as shown in Figure 8, there are multiple process variables, and the collected process variables in silicon single-crystal growth have different impacts on key variables. If too many input variables are used, it can lead to overfitting problems. Therefore, it is necessary to select input variables to obtain a more suitable subset for modeling. In this study, the Support Vector Machine Recursive Feature Elimination (SVM-RFE) algorithm was adopted for feature selection. The advantage of the SVM-RFE algorithm lies in considering the correlation and nonlinear relationship between features based on the idea of Support Vector Machines. By iteratively eliminating the least relevant features, this algorithm can better capture the correlation and importance between features. The available features are shown in Table 1. After feature selection, the retained feature indices are 1, 2, 3, 4, 6, 7, 10, and 11. We consider these 8 variables as the original features and provide the raw data of these 8 variables, as shown in Figure 9.

It is worth noting that, due to the difficulty in directly measuring temperature gradients during the actual growth process, the temperature gradient data used in this study are sourced from professional silicon single-crystal growth simulation software. It is widely believed in industry that the computational results of this simulation software are nearly identical to the actual results in industrial production processes. Moreover, there have been numerous studies utilizing this simulation software for data acquisition and fitting to further obtain the required data [26,27,28]. Therefore, the data acquisition process in this study can be considered reasonable.

### 4.2. Results and Discussion

The classification time interval for the thermal field temperature gradient change trend is set to 20 min. After processing, we obtained a total of 1830 sets of data. Among them, we selected 1200 sets of process data corresponding to the silicon content change trend as the training dataset, and 630 sets of process data as the test dataset.

To evaluate the performance of the MF-SAE-DBN method, we compared it with four other methods: PCA-SVM, SAE-SVM, PCA-RNN, and SAE-RNN. In PCA-SVM and PCA-RNN, we used Principal Component Analysis (PCA) to extract features and established classification models using Support Vector Machines (SVMs) and Recurrent Neural Networks (RNNs). In SAE-SVM, we used Stacked Autoencoders (SAEs) to extract deep-level features and then performed trend classification using SVM and RNN. It is worth noting that, except for PCA-SVM and PCA-RNN, all other models input multi-level fusion features.

The model parameter settings for each algorithm are detailed in Table 2.

**Figure 10:**
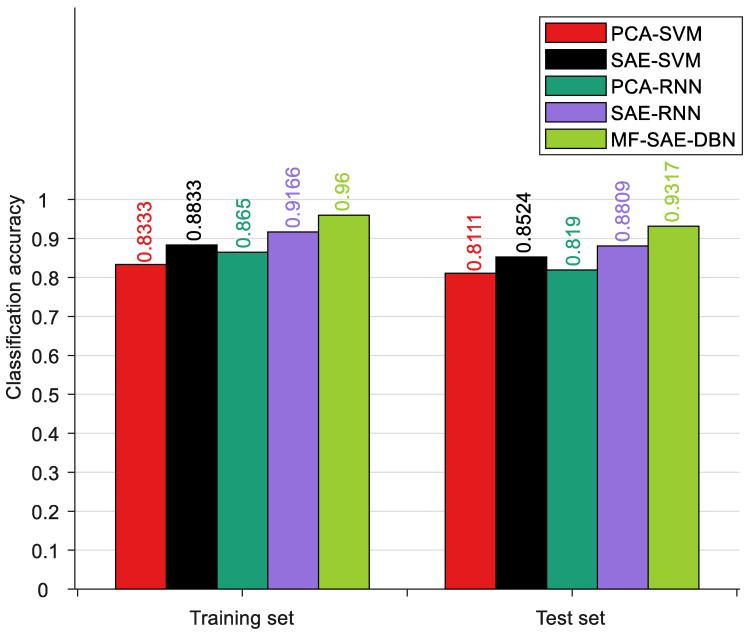
The classification results of different methods.

Evaluate the learning ability and classification ability of the aforementioned method using accuracy rate $h_{acc}$:

(16) $$h_{acc}=\sum_{i=1}^{n}\theta_{i}/n\times 100\%$$

where *n* is the number of test samples. When the *i*th sample is correctly classified, $\theta_{i}$ is equal to 1; otherwise, it is 0. All methods were implemented on the MATLAB R2020b platform. The accuracy is the average of the results from five cross-validation experiments. Please refer to Table 3 and Figure 10 for more specific details.

The research results indicate that among the trend classification algorithms, PCA-SVM has the lowest classification accuracy, followed by PCA-RNN. This is because these two algorithms only consider a single type of feature and the traditional PCA algorithm cannot guarantee the extraction of deep-level information contained in the data. In comparison, the RNN has improved feature mining capabilities compared to SVM, resulting in a slight improvement in the classification accuracy of PCA-RNN. On the other hand, SAE-SVM, SAE-RNN, and the proposed MF-SAE-DBN algorithm consider multi-level features, resulting in a higher classification accuracy than single-level features. Additionally, the combination of deep learning algorithms SAE and DBN significantly improves the predictive accuracy. This indicates the importance of considering multi-level features and the application of deep learning algorithms for trend classification tasks. The combination of SAE and DBN can better explore the latent features in the data and improve the predictive capability of the model. In summary, the proposed MF-SAE-DBN algorithm can accurately identify temperature gradient trend changes, provide an early warning for potential faults, and provide basic assurance for the safe and effective operation of equipment.

**Figure 11:**
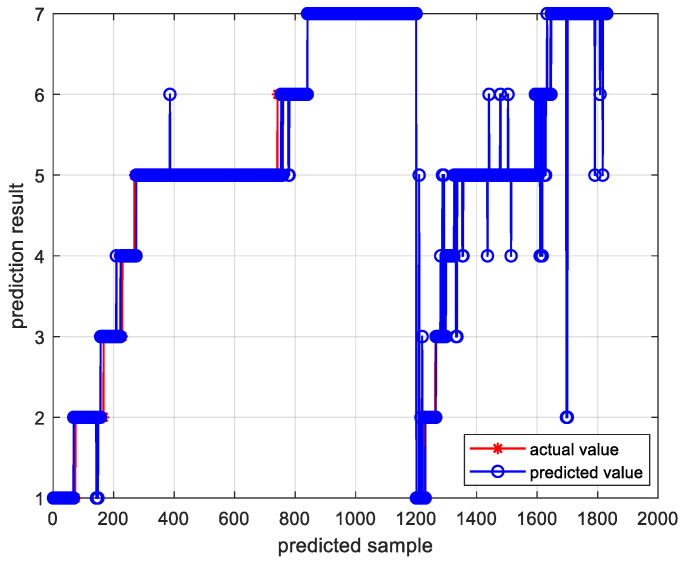
Trend prediction results of the proposed model.

**Figure 12:**
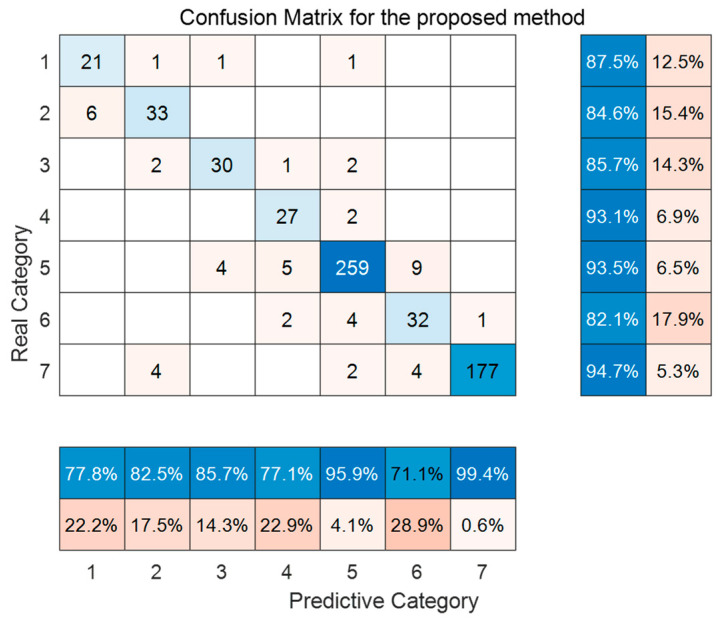
Confusion matrix of the proposed trend classification method.

Figure 11 and Figure 12, respectively, present the trend prediction results of the proposed model in this paper and the multi-class confusion matrix corresponding to the test dataset. In these figures, 1, 2, 3, 4, 5, 6, and 7 represent the convex decreasing trend, linear decreasing trend, concave decreasing trend, stable trend, convex increasing trend, linear increasing trend, and concave increasing trend, respectively. As shown in Figure 11, the first 1200 entries are the prediction results of the training dataset, while the remaining 630 entries are the prediction results of the test dataset. Combining with Figure 12, it can be observed that most trends in the test dataset are correctly classified. Out of 630 test samples, 579 were classified correctly, resulting in an overall accuracy of 91.90% for the model. The accuracy for the stable trend is 93.1%, and the hit rate for changing conditions exceeds 80%, which is valuable for practical industrial scenarios. In the actual growth process of silicon single crystals, maintaining stable operation and reasonable changes in thermal field temperature gradients is an essential requirement for ensuring crystal quality. In most cases, trend information does not change rapidly. Therefore, the accurate classification of stable conditions is crucial for providing guidance and decision-making information to on-site operators.

## 5. Conclusions

This paper proposes a silicon single-crystal growth temperature gradient trend classification algorithm based on multi-level feature fusion, aiming to accurately identify temperature gradient changes during silicon single-crystal growth and respond quickly to early fault information in crystal growth, ensuring the stable production of high-quality silicon single crystals. Firstly, the algorithm reasonably divides the temperature gradient trend based on expert knowledge and qualitative analysis methods. Secondly, it performs multi-level feature fusion on the original data features, shallow features, and deep abstract features, which are used as inputs to the classification network and which output seven different trend changes. The shallow features extract statistical characteristics of trend information, the deep features are extracted using the SAE algorithm, and the classification network uses the DBN. In the process of feature fusion, the influence of different features on the target variable is considered, and mutual information is calculated based on the difference between information entropy and conditional entropy, which is ultimately used for feature weighting. Compared with other algorithms, the proposed MF-SAE-DBN algorithm has better generalization and robustness, effectively classifies temperature gradient trends, and provides guidance information in advance to ensure the smooth growth of high-quality silicon single crystals. However, the algorithm also has certain limitations. Firstly, due to differences in lag parameters of heat fields of different sizes, the selection of time intervals in the trend division process is difficult to apply to all situations and needs to be adjusted according to the actual conditions. Secondly, although the algorithm has made progress in the research stage, overcoming some challenges is still necessary for its practical application in production.

To address these limitations, we believe it is necessary to further improve the algorithm. Firstly, we can enhance the classification accuracy by optimizing the algorithm’s parameters and adjusting the selection of time intervals. Secondly, exploring the use of more advanced network structures or employing other deep learning models can improve model training speed and accuracy. Additionally, considering the introduction of other relevant features or data sources to enhance the algorithm’s robustness and reliability is also a potential direction for our future efforts.

## Figures and Tables

**Table 1:** Variable description.

Ranking	Variable Name	Unit	Ranking	Variable Name	Unit
1	Heating Element Temperature	K	8	Secondary Heating Power	Kw
2	Liquid Surface Temperature	K	9	Crystal Ascending Speed	mm/min
3	Main Heater Power	Kw	10	Crucible Ascending Speed	mm/min
4	Crystal Diameter	mm	11	Crystal Weight	Kg
5	Average Growth Rate	mm/min	12	Magnetic Field Position	mm
6	Crystal Rotation Speed	rpm	13	Liquid Surface Position	mm
7	Crucible Rotation Speed	rpm	14	Heating Element Resistance	Ω

**Table 2:** Model parameter settings.

Models	Model Parameters
PCA-SVM	Kernel Function: Sigmoid, Regularization Parameter: 0.2
SAE-SVM	SAE Hidden Layer Node Numbers: [12, 18, 5], SAE Learning Rate: 0.01, Kernel Function: Sigmoid, Regularization Parameter: 0.2
PCA-RNN	RNN Hidden Layer Node Number: 50, RNN Learning Rate: 0.03
SAE-RNN	SAE Hidden Layer Node Numbers: [12, 18, 5], SAE Learning Rate: 0.01, RNN Hidden Layer Node Number: 50, RNN Learning Rate: 0.03
MF-SAE-DBN	SAE Hidden Layer Node Numbers: [12, 18, 5], SAE Learning Rate: 0.01, DBN Learning Rate: 0.1, RBM Iterations: 500, Hidden Layer Node Numbers: [7, 12].

**Table 3:** Trend classification accuracy of different methods.

Method	*h_train_*	*h_test_*
PCA-SVM	83.33%	81.11%
SAE-SVM	88.33%	85.24%
PCA-RNN	86.5%	81.9%
SAE-RNN	91.66%	88.09%
MF-SAE-DBN	96.00%	93.17%

## Data Availability

The research data is confidential in the industry. Due to the principle of confidentiality, the data cannot be shared. Please understand.

## Abbreviations

CZ	Czochralski
DBN	Deep Belief Network
FEM	Finite element method
SAE	stacked autoencoder
VMD	variational mode decomposition
SEAE	stacked enhanced autoencoder
TL	transfer learning
AE	Autoencoder
RBM	Restricted Boltzmann Machine
BP	backpropagation network
BPNN	backpropagation neural network
PCA	Principal Component Analysis
SVM	Support Vector Machine
RNN	Recurrent Neural Network
MF-SAE-DBN	Multi-level Feature fusion–Stacked Autoencoder–Deep Belief Network
Symbols	Designation
s	activation function
x	Input
$\hat{x}$	reconstructed input
W	network weights
b	network bias
J	loss function
E	energy function
P	probability distribution
R	range of variation
Δx/Δt	rate of change
S	standard deviation
Info(·)	information entropy
$G_{x}$	mutual information
$h_{acc}$	hit rate
